# Solução Custodiol®-HTK versus Cardioplegia Sanguínea Gelada em Cirurgia Coronária Isolada com Tempo de Pinçamento da Aorta Prolongado: Uma Análise de Propensão Pareada

**DOI:** 10.36660/abc.20190267

**Published:** 2020-08-19

**Authors:** Giuseppe Gatti, Pierpaolo Taffarello, Gabriella Forti, Carla Gripari, Gianfranco Gustin, Gianluca Castaldi, Ilaria Fiorica, Aniello Pappalardo

**Affiliations:** 1 Università degli Studi di Trieste Trieste Itália Università degli Studi di Trieste, Trieste – Itália

**Keywords:** Revascularização Miocárdica/complicações, Parada Cardíaca Induzida, Soluções Cardioplégicas/uso terapêutico, Reperfusão Miocárdica, Complicações Pós –Operatórias, Infarto do Miocárdio, Acidente Vascular Cerebral, Mortalidade

## Abstract

**Fundamento:**

A cardioplegia com solução Custodiol®-HTK (histidina-triptofano-cetoglutarato) é amplamente utilizada.

**Objetivo:**

Comparar os desfechos de cirurgia coronária isolada, com tempo de pinçamento da aorta (TPA) prolongado, em pacientes que receberam dose única de HTK ou repetidas doses de cardioplegia sanguínea gelada (MCB).

**Métodos:**

O TPA foi de ≥120 minutos para 148 pacientes consecutivos submetidos à cirurgia coronária isolada (2009−2016). A cardioplegia sanguínea gelada e a HTK foram utilizadas em 38 e 110 casos, respectivamente. Os dois grupos foram comparados considerando-se as características basais, os dados operatórios e os desfechos precoces. Uma vez que o perfil de risco e os dados operatórios diferiram significativamente entre os grupos, foi realizada análise utilizando pareamento por escore de propensão, sendo gerados 34 pares.

**Resultados:**

Enquanto o risco operatório esperado foi maior no grupo HTK, quando comparado com o grupo MCB (EUROSCORE II, p=0,005), não houve diferença significativa intergrupo em relação à mortalidade hospitalar (p=0,573). O balanço hídrico acumulado (positivo) no período pós-operatório (p=0,003), o número de transfusões de sangue (p=0,017), as taxas de lesão renal aguda (p=0,002) e qualquer complicação maior (p=0,019) foram mais elevados nos pacientes do grupo HTK. Todos esses resultados foram confirmados mesmo após o pareamento por escore de propensão, embora a diferença tenha sido significativa apenas em relação ao balanço hídrico (p=0,013) e bastante significativa em relação às transfusões de sangue (p=0,054). No grupo HTK, o tempo de permanência hospitalar e na unidade de terapia intensiva foram mais longos tanto para a amostra global (p=0,016 e 0,008) quanto para a amostra pareada (p=0,142 e 0,198). Na amostra pareada, os valores pico para troponina I cardíaca sérica foram mais baixos no grupo HTK (p=0,122); os níveis séricos de creatinina foram mais baixos no grupo MCB (p=0,023).

**Conclusão:**

Para os pacientes deste estudo que necessitaram de TPA prolongado, houve uma tendência a desfechos piores quando a cardioplegia com HTK, e não a cardioplegia sanguínea gelada, foi utilizada. (Arq Bras Cardiol. 2020; 115(2):241-250)

## Introdução

A Solução Custodiol®-HTK (histidina-triptofano-cetoglutarato) (Pharma, Newtown, PA, USA) é classificada como uma solução cardioplégica cristaloide intracelular devido à sua baixa concentração de sódio e cálcio.^1^ A depleção de sódio do espaço extracelular causa a hiperpolarização da membrana plasmática do miócito, induzindo parada cardíaca na diástole. A alta concentração de histidina atenua a acidose causada pela acumulação de metabolitos anaeróbicos durante o período de isquemia; o cetoglutarato melhora a produção de adenosina trifosfato durante a reperfusão; o triptofano estabiliza a membrana celular.^2^ Alega-se que uma única dose de cardioplegia oferece proteção por um período de até 3 horas.^2 , 3^ Consequentemente, ela é geralmente utilizada para a proteção do miocárdio durante cirurgias cardíacas complexas, seja em adultos^4 , 5^ ou crianças,^6^ e para a preservação nas cirurgias de transplante.^7 , 8^ De fato, o tempo de pinçamento da aorta (TPA) seguro, utilizando-se a cardioplegia com solução Custodiol®-HTK, ainda não foi determinado. Além disso, há preocupações em relação à hiponatremia que sucede a rápida administração do grande volume necessário desta solução cardioplégica com baixa concentração de sódio,^9 , 10^ bem como à eficácia da proteção miocárdica promovida pela técnica de cardioplegia de dose única.^7 , 8 , 11^ Ademais, apesar de ser amplamente utilizada, poucos estudos clínicos compararam a solução Custodiol®-HTK com a cardioplegia tradicional, sanguínea ou cristaloide, na cirurgia de revascularização da artéria coronária.^12^_._

O objetivo deste estudo retrospectivo foi comparar a cardioplegia utilizando solução Custodiol®-HTK e a cardioplegia sanguínea gelada (MCB) de múltiplas doses em pacientes submetidos à cirurgia coronária isolada, quando o TPA prolongado se faz necessário. Tanto a proteção miocárdica quanto o desfecho precoce pós-operatório foram examinados.

## Métodos

Entre julho de 2009 e outubro de 2016, a cardioplegia com Custodiol®-HTK foi utilizada em 106 pacientes consecutivos submetidos à revascularização coronária isolada, no Departamento de Cirurgia Cardíaca do Hospital Universitário de Trieste, Italy; para 38 (35,8%) deles, o TPA foi ≥120 minutos. Os desfechos pós-operatórios precoces desses 38 pacientes (grupo HTK) foram comparados com aqueles de 110 pacientes consecutivos, submetidos à cirurgia coronária na cidade de Trieste, durante o mesmo período, cujo TPA foi ≥120 minutos, e que receberam cardioplegia sanguínea gelada (grupo MCB). Uma vez que os dois grupos apresentaram resultados significativamente diferentes no que diz respeito ao perfil de risco e aos dados cirúrgicos, também foi realizada uma análise de propensão pareada.

Salvo disposto em contrário, as definições e os valores de corte das variáveis pré-operatórias foram aqueles utilizados no Sistema Europeu para Avaliação de Risco em Cirurgia Cardíaca (EuroSCORE II).^13^ O perfil de risco de cada paciente foi estabelecido antes da cirurgia, conforme o EuroSCORE II. As definições de complicações pós-operatórias estavam em consonância com as definições internacionalmente acordadas sobre complicações pós cirurgia cardíaca.^14^ As disfunções neurológicas permanentes (AVC com lesões focais detectadas através de tomografia computadorizada), ventilação invasiva prolongada (>48 h), infarto do miocárdio, baixo débito cardíaco (requerendo altas doses de agentes inotrópicos, uso de balão intra-aórtico ou de oxigenador de membrana extracorpórea), lesão renal aguda (com ou sem terapia renal substitutiva), falência de múltiplos órgãos, múltiplas transfusões de sangue (três ou mais unidades de concentrado de hemácias), reexploração mediastinal para sangramento ou tamponamento, infecção profunda da ferida esternal (infecção incisional profunda ou mediastinite) e sepse foram definidas como complicações maiores. Mortalidade hospitalar e complicações maiores foram incluídas no desfecho combinado.

### Protocolos de Administração de Cardioplegia

A via de administração da solução Custodiol®-HTK e da cardioplegia sanguínea foram sempre a anterógrada e retrógrada, à temperatura de ~4°C. Cada paciente recebeu 20-25 ml de solução HTK por kg de peso corporal. A pressão de perfusão na raiz da aorta foi mantida constante em 100 mmHg até a parada cardíaca e, em seguida, entre 40–50 mmHg. Durante a perfusão retrógrada, a pressão do seio coronário manteve-se entre 20 mmHg e 25 mmHg. O tempo acumulado de perfusão variou entre 6-8 minutos. Na presença de hiponatremia, foi utilizada solução de cloreto de sódio (3 mEq/ml) para correção; a hipotensão sistêmica foi tratada com infusão de norepinefrina. A hemodiluição foi mitigada pela remoção de parte da solução HTK da raiz da aorta durante a perfusão retrógrada.^5 , 7 , 8^ A cardioplegia sanguínea tradicional (cardioplegia de Buckberg) foi administrada a cada 20 minutos, conforme protocolos padrão.^15^

### Exames de sangue

Os níveis sanguíneos de hemoglobina, creatinina, sódio, potássio e cálcio foram medidos antes da cirurgia e imediatamente após a internação do paciente na unidade de terapia intensiva; a contagem de plaquetas e o perfil de coagulação sanguínea também foram examinados antes e depois da cirurgia. Os níveis séricos de creatina quinase, creatina quinase-MB, troponina I cardíaca e aspartato aminotransferase foram medidos durante a permanência dos pacientes na UTI. Foram comparados os valores entre as amostras pareadas dos grupos HTK e MCB.

Os pacientes receberam informações sobre o estudo, mas foram dispensados de fornecer consentimento individual, conforme a legislação italiana. Embora envolva seres humanos, este estudo retrospectivo não foi registrado em um banco de dados acessível ao público.

### Métodos estatísticos

As variáveis contínuas com distribuição normal foram expressas como média ± desvio-padrão e aquelas sem distribuição normal, como mediana e o intervalo entre o primeiro e o terceiro quartil. As variáveis categóricas foram expressas como frequências e percentuais. A comparação estatística das características basais dos pacientes, os dados operatórios e as complicações pós-operatórias foi realizada utilizando-se o teste qui-quadrado ou o teste exato de Fisher para as variáveis categóricas, e o teste t de Student não pareado ou o teste U de Mann-Whitney, para variáveis contínuas. Uma vez que os grupos HTK e MCB diferiram significativamente em relação ao perfil de risco, ao número de anastomoses entre as artérias coronárias, e ao TPA, uma análise multivariada foi realizada pelo uso da técnica *stepwise* na regressão logística. A área sob a curva (característica de operação do receptor), com um intervalo de confiança (IC) de 95%, foi utilizada para representar a probabilidade de regressão. Para estimar a probabilidade de cada paciente ser alocado em um ou em outro grupo, um escore de propensão (EP) foi calculado por um modelo não parcimonioso, considerando as seguintes características pré-operatórias dos pacientes: idade, sexo, hipertensão, índice de massa corporal, hemoglobina, diabetes controlada com insulina, doença pulmonar crônica, taxa de filtração glomerular estimada pela fórmula de Cockcroft-Gault, diálise crônica, arteriopatia extracardíaca, classe funcional IV da New York Heart Association (NYHA), Angina classe 4 (CCS), infarto do miocárdio recente, doença da artéria coronária esquerda, número de vasos coronários acometidos, fração de ejeção do ventrículo esquerdo, uso de balão intra-aórtico, prioridade cirúrgica, risco operatório esperado de acordo com o EuroSCORE II, número de anastomoses entre as artérias coronárias, e a duração do TPA. O pareamento por EP foi aplicado utilizando-se o pareamento por vizinho mais próximo e o pareamento por *caliper* (DP=0,2) do modelo *logit* do EP estimado. Para avaliar o equilíbrio entre as amostras pareadas foram utilizados o teste de McNemar, para variáveis dicotômicas, o teste t de Student, para amostras pareadas, ou o teste de Wilcoxon, para variáveis contínuas, e a análise das diferenças padronizadas após o pareamento. Diferenças padronizadas <10% foram consideradas um desequilíbrio aceitável entre os grupos de tratamento. Os mesmos testes foram adotados para estimar as diferenças nos dados operatórios e nas complicações pós-operatórias das amostras pareadas. A análise de variância de duas vias foi utilizada para observar a interação entre o tipo de cardioplegia e qualquer complicação maior para características basais relevantes entre as amostras pareadas por EP. Todos os testes foram bicaudais com o valor de p fixado em 0,05 para significância estatística. A inserção de dados foi realizada utilizando-se o Microsoft Office Excel, versão 2007. A análise dos dados foi realizada no programa SPSS for Windows, versão 13.0 (SPSS, Inc., Chicago, IL, USA).

## Resultados

### Amostra global

Embora os grupos HTK e MCB tenham apresentado diferenças significativas quanto ao risco operatório esperado de acordo com o EuroSCORE II (p=0,005), número total de anastomoses coronárias (p=0,003) e TPA (p=0,031), não foram observadas diferenças estatisticamente significativas no que diz respeito à mortalidade hospitalar (p=0,573). Entretanto, o balanço hídrico acumulado (positivo) no período pós-operatório (p=0,003), lesão renal aguda (p=0,002), número de unidades de concentrado de hemácias transfundidas (p=0,017), e taxa global de complicações maiores (p=0,019) foram significativamente mais altas nos pacientes do grupo HTK que, consequentemente, apresentaram tempo de internação maior (p=0,008) ( Tabelas 1-3 ).

**Tabela 1:** – Características basais e perfis de risco dos pacientes*,†

Características	Amostra global				Amostra pareada por EP			

	Grupo HTK n=38	Grupo MCB n=110	Valor de p	Diferença padronizada	Grupo HTK n=34	Grupo MCB n=34	Valor de p	Diferença padronizada
Idade (anos)	66±9,5	66,3±9	0,850		66,1±10	65,3±9,6	0,740	
70–80	12 (31,6)	40 (36,4)		0,001	12 (35,3)	12 (35,3)		0,027
>80	2 (5,3)	5 (4,5)		0,018	2 (5,9)	1 (2,9)		0,010
Sexo feminino	4 (10,5)	11 (10)	1,000	0,057	2 (5,9)	1 (2,9)	1,000	0,001
Hipertensão	32 (84,2)	85 (77,3)	0,489	0,007	28 (82,4)	24 (70,6)	0,252	0,000
Índice de massa corporal, kg/m^2^	28±5,1	27,6±3,7	0,582		28,2±5,1	27,2±3,1	0,318	
>30	10 (26,3)	26 (23,6)		0,008	9 (26,5)	9 (26,5)		0,001
Anemia‡	18 (47,4)	43 (39,1)	0,371	0,010	16 (47,1)	18 (52,9)	0,624	0,000
Diabetes controlada com insulina	5 (13,1)	7 (6,4)	0,298	0,007	3 (8,8)	5 (14,7)	0,709	0,003
Doença pulmonar crônica	4 (10,5)	8 (7,3)	0,731	0,018	3 (8,8)	3 (8,8)	1,000	0,005
TFGe, ml/min§	75,1±39	83,5±28,4	0,156		74,9±40,9	78,6±26,7	0,659	
50–85	12 (31,6)	44 (40)		0,032	10 (29,4)	13 (38,2)		0,001
≤50	10 (26,3)	11 (10)		0,018	9 (26,5)	6 (17,6)		0,003
Diálise crônica	2 (5,3)	2 (1,8)	0,573	0,131	2 (5,9)	1 (2,9)	1,000	0,014
Arteriopatia extracardíaca	13 (34,2)	27 (24,5)	0,247	0,015	12 (35,3)	10 (29,4)	0,603	0,001
NYHA classe IV	2 (5,3)	4 (3,6)	1,000	0,092	2 (5,9)	3 (8,8)	1,000	0,012
CCS classe 4	24 (63,2)	52 (47,3)	0,091	0,026	20 (58,8)	18 (52,9)	0,624	0,002
Infarto do miocárdio recente	7 (18,4)	21 (19,1)	0,920	0,034	6 (17,6)	5 (14,7)	0,740	0,006
Doença arterial coronariana			0,423				0,606	
Lesão de dois vasos	1 (2,6)	9 (8,2)		0,084	1 (2,9)	3 (8,8)		0,004
Lesão de três vasos	37 (97,4)	101 (91,8)		0,008	33 (97,1)	31 (91,2)		0,000
Artéria coronária esquerda	10 (26,3)	43 (39,1)	0,156	0,042	10 (29,4)	10 (29,4)	1,000	0,006
FEVE, %	52,2±13	54,9 ± 10,1	0,205		52±12,7	52,1±11,4	0,960	
31–50	13 (34,2)	30 (27,3)		0,012	11 (32,4)	13 (38,2)		0,001
<31	2 (5,3)	4 (3,6)		0,060	2 (5,9)	2 (5,9)		0,008
Uso de BIA	5 (13,1)	11 (10)	0,762	0,016	3 (8,8)	2 (5,9)	1,000	0,003
Prioridade Cirúrgica			0,275				0,858	
Eletiva	8 (21,1)	37 (33,6)		0,024	8 (23,5)	10 (29,4)		0,006
Urgente	29 (76,3)	72 (65,5)		0,031	25 (73,5)	23 (67,6)		0,003
Emergência	1 (2,6)	1 (0,9)		-	1 (2,9)	1 (2,9)		-
Risco operatório esperado	3,5 (1,6−6,2)	1,7 (1,1−3,9)	0,005		3,3 (1,5−5,8)	3 (1,4−4,8)	0,812	
(EuroSCORE II), %||								

*As variáveis contínuas foram expressas como média ± DP, ou mediana e o intervalo entre o primeiro e o terceiro quartil. As variáveis categóricas foram expressas como frequências e percentuais. †Salvo disposto em contrário, as definições e os valores de corte das variáveis pré-operatórias foram aqueles utilizados no EuroSCORE II. ‡Definida como hemoglobina <13 g/dl para homens e <12 g/dl para mulheres. § A taxa de depuração da creatinina, calculada pela fórmula Cockcroft-Gault, foi utilizada para aproximação da TFG. ||Ref, 13. CCS: Sociedade Cardiovascular Canadense; TFGe: taxa de filtração glomerular estimada; EuroSCORE: Sistema Europeu para Avaliação de Risco em Cirurgia Cardíaca; HTK: histidina-triptofano-cetoglutarato; BIA: balão intra-aórtico; FEVE: fração de ejeção do ventrículo esquerdo; MCB: cardioplegia sanguínea gelada multidose; NYHA: New York Heart Association; EP: escore de propensão; DP: desvio-padrão.

### Amostras pareadas por EP

O escore de propensão foi estimado por meio de regressão logística e a área sob a curva ROC foi de 0,75 (95 %IC, 0,67–0,81). O emparelhamento individual resultou em 34 pares de pacientes HTK/MCB com características basais, perfis de risco e dados operatórios semelhantes, o que pode ser confirmado pelo fato de todas as diferenças padronizadas terem ficado abaixo de 10% (Tabelas 1 e 2). O balanço hídrico acumulado (positivo) no período pós-operatório foi significativamente maior no grupo HTK quando comparado com o grupo MCB (p=0,013) ( Tabela 2 ). No grupo HTK, registrou-se uma tendência a um número maior de unidades de concentrado de hemácias transfundidas (p=0,054), aumento do risco de lesão renal aguda (p=0,150) e qualquer complicação maior (p=0,128), bem como maior tempo de permanência na unidade de terapia intensiva (p=0,142) e tempo de internação hospitalar (p=0,198) ( Tabela 3 ). O teste de interação mostrou que o uso da solução Custodiol®-HTK nos pacientes com comprometimento renal pode aumentar o risco de qualquer complicação pós-operatória (p=0,183; Tabela 4 ). Entre os dois grupos, não houve diferenças significativas nas alterações dos níveis sanguíneos de hemoglobina, plaquetas e eletrólitos. Do mesmo modo, nenhuma alteração significativa foi observada no perfil de coagulação entre os valores registrados imediatamente antes da cirurgia e aqueles obtidos imediatamente após a internação dos pacientes na UTI, ao passo que as diferenças nos níveis de creatinina foram significativas (p=0,023; Figura 1 ). Finalmente, os valores pico para troponina I cardíaca foram mais baixos no grupo HTK do que no grupo MCB, embora a diferença não tenha sido muito significativa (p=0,122; Figura 2 ).

**Tabela 2:** – Dados operatórios*

Dados	Amostra global				Amostra pareadas por EP			

	Grupo HTK n=38	Grupo MCB n=110	Valor de p	Diferença padronizada	Grupo HTK n=34	Grupo MCB n=34	Valor de p	Diferença padronizada
No. de anastomoses coronárias	5,2±1,3	4,6±1	0,003		5,1±1	5,2±1	0,481	
Enxerto de ATI			0,249				0,510	
Bilateral	30 (78,9)	97 (88,2)			27 (79,4)	30 (88,2)		
Unilateral	8 (21,1)	12 (10,9)			7 (20,6)	4 (11,8)		
EVS isolado	0	1 (0,9)			0	0		
TPA, min	136 (128−148)	130 (124−139)	0,031		137 (128−148)	135,5 (125,5−145,5)	0,971	
120–149	29 (76,3)	97 (88,2)		0,001	26 (76,5)	27 (79,4)		0,000
150–179	8 (21,1)	10 (9,1)		0,022	8 (23,5)	5 (14,7)		0,002
180–209	0	3 (2,7)		0,050	0	2 (5,9)		0,007
>209	1 (2,6)	0		-	0	0		-
TV/FV após a	2 (5,3)	12 (10,9)	0,362		0	0	-	
liberação da pinça da aorta								
Duração da CEC (min)	234 (217−259)	225 (209−253)	0,193		224,5 (216−252)	234,5 (222−261,5)	0,464	
Duração da cirurgia (min)	345 (326−389)	340 (315−369)	0,412		341 (326−378,5)	338 (320−366,5)	0,962	
Balanço hídrico acumulado/ASC (ml/m^2^)	+2900 (2440−3590)	+2440 (2030−3140)	0,003		+2860 (2440−3660)	+2500 (2000−3030)	0,013	

* As variáveis contínuas foram expressas como média ± DP, ou mediana e o intervalo entre o primeiro e o terceiro quartil. As variáveis categóricas foram expressas como frequências e percentuais. ASC: área da superfície corporal; CEC: circulação extracorpórea; HTK: histidina-triptofano-cetoglutarato; ATI: artéria torácica interna; MCB: cardioplegia sanguínea gelada multidose; EP: escore de propensão; DP: desvio-padrão; EVS: enxerto de veia safena; TV/FV: taquicardia/fibrilação ventricular; TPA: tempo de pinçamento da aorta.

**Tabela 3:** – Desfechos hospitalares*,†

Complicação	Amostra global			Amostra pareada por EP		

	Grupo HTK n=38	Grupo MCB n=110	Valor de p	Grupo HTK n=34	Grupo MCB n=34	Valor de p
Mortalidade hospitalar/30 dias	2 (5,3)	2 (1,8)	0,573	1 (2,9)	0	1,000
Disfunção neurológica (qualquer)	2 (5,3)	8 (7,3)	1,000	2 (5,9)	5 (14,7)	0,427
Transitória‡	1 (2,6)	8 (7,3)	0,448	1 (2,9)	5 (14,7)	0,197
Permanente (AVC)	1 (2,6)	2 (1,8)	1,000	0	1 (2,9)	1,000
Ventilação invasiva prolongada (>48 h)	3 (7,9)	6 (5,5)	0,695	2 (5,9)	3 (8,8)	1,000
Fibrilação atrial, recente	6 (15,8)	16 (14,5)	0,862	5 (14,7)	4 (11,8)	1,000
Infarto do miocárdio	0	1 (0,9)	1,000	0	1 (2,9)	1,000
Disfunção do VD	4 (10,5)	8 (7,3)	0,731	4 (11,8)	1 (2,9)	0,356
Baixo débito cardíaco	6 (15,8)	10 (9,1)	0,362	3 (8,8)	3 (8,8)	1,000
Terapia inotrópica prolongada (>12 h)	23 (60,5)	57 (51,8)	0,354	19 (55,9)	19 (55,9)	1,000
Uso do BIA no intra e pós-operatório	0	9 (8,2)	0,112	0	3 (8,8)	0,239
Lesão renal aguda	8 (21,1)	4 (3,6)	0,002	7 (20,6)	2 (5,9)	0,150
Terapia renal substitutiva	5 (13,2)	0	0,001	4 (11,8)	0	0,114
Falência de múlitplos órgãos	3 (7,9)	3 (2,7)	0,339	2 (5,9)	1 (2,9)	1,000
Débito do dreno torácico de 48h/ASC, ml/m2	685 (390–1074,5)	551 (372,5–970)	0,463	616,5 (390–1074,5)	633 (381–953,5)	0,609
No. de unidades de CH	1,5 (1–3)	1 (0–2)	0,017	2 (1–3)	1 (0–2)	0,054
Múltiplas transfusões (>2 unidades de CH)	12 (31,6)	19 (17,3)	0,062	11 (32,4)	7 (20,6)	0,271
Transfusões maciças (>4 unidades de CH)	5 (13,2)	2 (1,8)	0,012	4 (11,8)	0	0,114
Reexploração mediastinal	4 (10,5)	6 (5,5)	0,454	3 (8,8)	2 (5,9)	1,000
Infecção profunda da ferida esternal	1 (2,6)	4 (3,6)	1,000	0	1 (2,9)	1,000
Sepse	0	2 (1,8)	1,000	0	1 (2,9)	1,000
Qualquer complicação maior§	17 (44,7)	27 (24,5)	0,019	15 (44,1)	9 (26,5)	0,128
Tempo de internação (dias)	13,5 (10–18)	10 (8–15)	0,008	14 (10–19)	10 (8–15)	0,198
Tempo na UTI (dias)	3 (2–5,5)	2 (1,5–3)	0,016	3 (2–5,5)	2 (1,5–3)	0,142

* As variáveis contínuas foram expressas como média ± DP, ou mediana e o intervalo entre o primeiro e o terceiro quartil. As variáveis categóricas foram expressas como frequências e percentuais. †Salvo disposto em contrário, as definições de complicações pós-operatórias estavam em consonância com as definições internacionalmente acordadas sobre complicações pós cirurgia cardíaca. ‡Inclusive retardo no despertar, distúrbios psiquiátricos manifestos e convulsões. §Inclusive mortalidade hospitalar, AVC, ventilação invasiva prolongada, infarto do miocárdio, baixo débito cardíaco, lesão renal aguda, falência de múltiplos órgãos, múltiplas transfusões sanguíneas, reexploração mediastinal, infecção profunda da ferida esternal e sepse. ASC: área da superfície corporal; HTK: histidina-triptofano-cetoglutarato; BIA: balão intra-aórtico; MCB: cardioplegia sanguínea gelada multidose; EP: escore de propensão; CH: concentrado de hemácias; VD: ventrículo direito.

**Tabela 4:** – Análise da taxa de qualquer complicação pós-operatória maior* em diferentes subgrupos de com teste de interação†

Características	Grupo HTK %	Grupo MCB %	OR	95% IC	Interação

					Valor de p
Global	44,1	26,5	2,19	0,79−6,08	0,132
Sem anemia	33,3	25	1,5	0,34–6,7	0,391
Anemia‡	56,2	27,8	3,34	0,8–13,9	
TFGe >85 ml/min§	33,3	33,3	1,00	0,22–4,56	0,183
TFGe ≤85 ml/min§	52,6	21,1	4,17	1–17,3	
CCS classes 1–3	50	31,2	2,2	0,5–9,75	0,970
CCS classe 4	40	22,2	2,33	0,56–9,72	
Infarto do miocárdio recente	46,4	27,6	2,27	0,76–6,85	0,864
Sem infarto do miocárdio recente	33,3	20	2	0,13–32	
FEVE >50%	38,1	26,3	1,72	0,45–6,64	0,522
FEVE ≤50%	53,8	26,7	3,21	0,66–15,6	

*Inclusive mortalidade hospitalar, AVC, ventilação invasiva prolongada, infarto do miocárdio, baixo débito cardíaco, lesão renal aguda, falência de múltiplos órgãos, múltiplas transfusões de sangue, reexploração mediastinal, infecção profunda da ferida esternal e sepse. † Salvo indicação em contrário, as definições e os valores de corte das variáveis pré-operatórias foram aqueles utilizados no EuroSCORE II. ‡Definida como hemoglobina <13 g/dl para homens e <12 g/dl para mulheres. § A taxa de depuração da creatinina, calculada pela fórmula Cockcroft-Gault, foi utilizada para aproximação da TFG. CCS: Sociedade Cardiovascular Canadense; IC=Intervalo de confiança; TFGe: taxa de filtração glomerular estimada; EuroSCORE: Sistema Europeu para Avaliação de Risco em Cirurgia Cardíaca; HTK: histidina-triptofano-cetoglutarato; FEVE: Fração de Ejeção do Ventrículo Esquerdo; MCB: cardioplegia sanguínea gelada multidose; OR: razão de probabilidade.

**Figura 1A:**
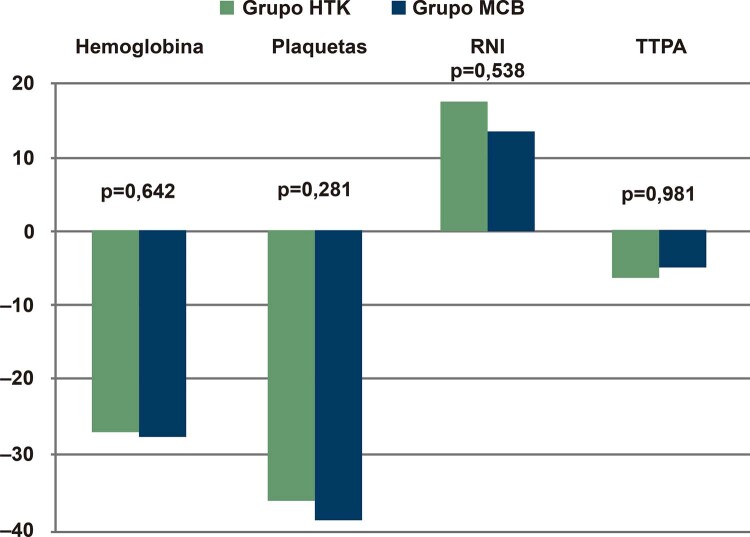
– *Grupo HTK vs. Grupo MCB. Amostras pareadas por EP. Diferenças nas alterações dos níveis sanguíneos de hemoglobina, plaquetas, RNI e TTPA entre os valores obtidos antes da cirurgia e imediatamente após a internação do paciente na UTI. TTPA: tempo de tromboplastina parcial ativada; HTK: histidina-triptofano-cetoglutarato; RNI: razão normalizada internacional; MCB; cardioplegia sanguínea gelada multidose; EP: escore de propensão.*

**Figura 1B:**
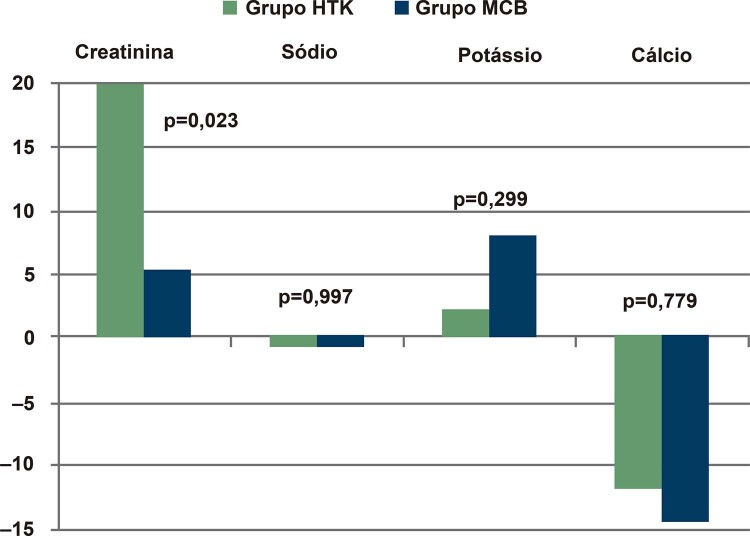
– *Grupo HTK versus Grupo MCB. Amostras pareadas por EP. Diferenças nas alterações dos níveis sanguíneos de creatinina, sódio, potássio e cálcio entre os valores obtidos antes da cirurgia e imediatamente após a internação do paciente na UTI. HTK: histidina-triptofano-cetoglutarato; MCB: cardioplegia sanguínea gelada multidose; EP: escore de propensão.*

**Figura 2:**
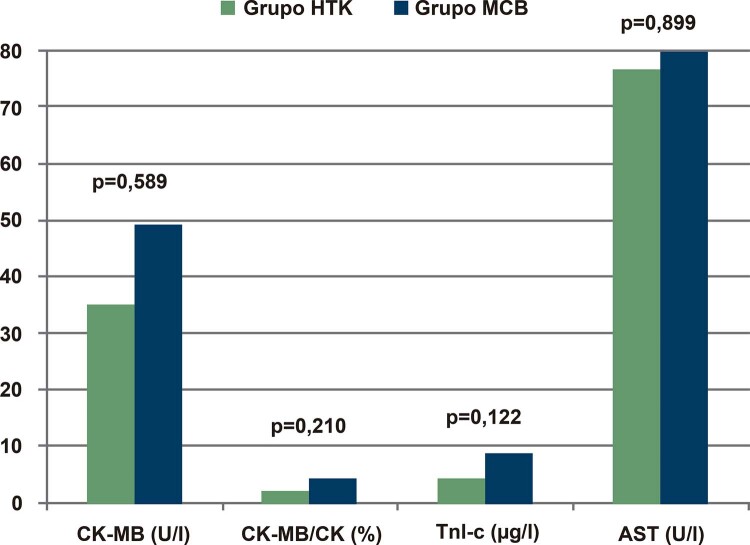
– *Grupo HTK versus Grupo MCB. Amostras pareadas por EP. Diferenças nos níveis séricos pico após a cirurgia de CK-MB, CK-MB/CK, TnI e AST. AST: aspartato aminotransferase; CK: creatina quinase; CK-MB: creatina quinase-MB; TnI-c: troponina cardíaca I; HTK: histidina-triptofano-cetoglutarato; MCB: cardioplegia sanguínea gelada multidose; EP: escore de propensão.*

## Discussão

O achado mais relevante deste estudo foi que, na cardioplegia sanguínea tradicional, a solução Custodiol®-HTK não melhorou os desfechos clínicos de uma amostra limitada de pacientes submetidos à cirurgia coronária isolada com TPA de 120 minutos ou mais. De fato, após o pareamento por propensão, foram observados níveis elevados de creatinina em pacientes do grupo HTK logo após a cirurgia, bem como uma tendência a um maior número de unidades de concentrado de hemácias transfundidas, aumento do risco de lesão renal aguda (e terapia renal substitutiva) e qualquer complicação maior, e tempos de permanência na UTI e de hospitalização maiores. Além disso, o teste de interação mostrou que o uso da solução Custodiol®-HTK nos pacientes com complicação renal pode aumentar o risco de qualquer complicação pós-operatória. No entanto, o pior desempenho dessa cardioplegia intracelular no subgrupo de pacientes de difícil manejo deste estudo não esteve relacionado à proteção miocárdica inadequada. Na realidade, entre os grupos HTK e MCB não houve diferenças nem nas taxas de infarto do miocárdio e baixo débito cardíaco, nem nas taxas de seus substitutos, tais como terapia inotrópica prolongada e uso do BIA no intra e pós-operatório. Além disso, os valores pico para troponina I cardíaca tenderam a ser mais baixos no grupo HTK quando comparado com o grupo MCB. A necessidade de aumento de unidades de concentrado de hemácias, principalmente durante a cirurgia, no grupo HTK deveu-se sobretudo à hemodiluição associada à infusão de cardioplegia cristaloide, em grandes volumes, conforme exigido por esse método. Apesar de parte da solução HTK ter sido removida da raiz da aorta durante a infusão de modo retrógrado, o balanço hídrico acumulado (positivo) no período pós-operatório foi, de fato, significativamente maior nos pacientes HTK do que nos pacientes do grupo MCB, até mesmo após a análise de propensão pareada. Na verdade, não houve aumento do sangramento pós-operatório, nem qualquer diferença no tocante aos perfis de coagulação sanguínea nos pacientes HTK, quando comparados com os pacientes MCB. Tanto a hemodiluição, quanto a necessidade de transfusões subsequentes que ocorreu no grupo HTK durante a operação, bem como o balanço hídrico acumulado (positivo) que se seguiu, poderiam explicar tanto o aumento na taxa de lesão renal aguda (e terapia renal substitutiva) nesses pacientes, quanto o aumento do risco de qualquer complicação pós-operatória nos pacientes do grupo HTK com comprometimento renal. Certamente, alguns efeitos metabólicos sistêmicos podem estar envolvidos. Por exemplo, algum grau de acidose metabólica frequentemente ocorre após o uso da solução de Custodiol®-HTK, que deve ser imediatamente neutralizado.^1 - 3^ Todavia, uma vez que não foram relatados dados perioperatórios referentes ao pH ou lactacidemia, essa hipótese não pôde ser confirmada neste estudo. Por outro lado, dado que a solução Custodiol®-HTK vem sendo utilizada com sucesso na preservação da função renal, na cirurgia de transplante de rim,^16^ a lesão renal direta parece improvável.

Embora tenha havido uma diferença evidente em relação à mortalidade hospitalar entre os dois grupos do estudo (5,6% versus 1,8%), essa diferença não foi significativa (p=0,573), talvez por conta do número limitado de pacientes do estudo. Entretanto, não houve diferença na mortalidade hospitalar (p=1,000) após o pareamento por propensão, o que compensou a diferença no risco operatório esperado na amostra global. Não houve registro de aumento de taquicardia ventricular espontânea ou fibrilação após a liberação do pinçamento da aorta. Nenhum aumento significativo do risco de disfunção ventricular direita foi relatado. O uso tanto da solução de Custodiol®-HTK quanto da cardioplegia sanguínea não trouxe benefícios para os pacientes com anemia, angina instável, infarto do miocárdio recente ou disfunção ventricular esquerda. O controle perioperatório rigoroso e eficaz da hiponatremia, que foi recebido pelos pacientes do estudo, poderia explicar a baixa taxa de qualquer disfunção neurológica transitória que tenha ocorrido no grupo HTK. A taxa mais baixa de quaisquer disfunções neurológicas transitórias observadas nos pacientes do grupo HTK em relação ao grupo MCB (embora a diferença não tenha sido muito significativa) foi um resultado inesperado para os autores deste estudo.

O papel da solução Custodiol®-HTK na cirurgia cardíaca em adultos ainda não foi explorado a fundo. Geralmente, os autores que investiga os desfechos subsequentes à cirurgia cardíaca minimamente invasiva utilizando solução Custodiol®-HTK concordam que evitar repetidas infusões pode diminuir o risco de má perfusão coronária devido ao deslocamento do clampeamento endoaórtico (se realizado) e aumentar o conforto do médico cirurgião durante o procedimento.^17 - 20^ Quase todos os pesquisadores que compararam o Custodiol®-HTK com a cardioplegia sanguínea gelada exibiram desfechos clínicos semelhantes para ambas as opções.^5 , 7 , 8 , 17 , 19^ De fato, apenas poucos estudos demonstraram alguns benefícios trazidos por uma ou outra estratégia cardioplégica. Por exemplo, Scrascia et al.,^4^ relataram valores baixos de troponina I cardíaca para um TPA >160 minutos em pacientes submetidos à cirurgia da aorta com Custodiol®-HTK. Prathanee et al.,^12^ criaram um estigma em torno do uso da cardioplegia com Custodiol®-HTK na cirurgia coronária isolada, ao afirmarem que ele levaria a um aumento significativo do risco de fibrilação ventricular após a liberação da pinça da aorta. Contudo, nenhum significado clínico foi correlacionado com esse fato. Muito recentemente, em uma amostra de 362 pacientes submetidos à cirurgia valvar cardíaca (minimamente invasiva ou aberta), Hummel et al.,^20^ demonstraram desfechos superiores nos pacientes tratados com HTK, em relação à transfusão de sangue, AVC e reinternação em 30 dias após a alta, o que se traduz em uma economia líquida média de aproximadamente US$ 3.000,00 por paciente. Finalmente, desfechos equivalentes entre o uso da solução Custodiol®-HTK e a cardioplegia sanguínea gelada foram observados por Hoyer et al.,^11^ em 825 amostras de pacientes pareados por escore de propensão, ainda que a cardioplegia sanguínea tenha se mostrado benéfica na disfunção ventricular esquerda.

Considerando-se que uma dose única de solução Custodiol®-HTK supostamente ofereceria proteção micárdica prolongada,^2 , 3^ ela foi geralmente utilizada durante o período de estudo, na cirurgia coronária, em pacientes com expectativa de cirurgia de longa duração (número elevado de anastomoses coronárias, estreitamento do diâmetro dos vasos coronários, curso intramiocárdico, lesões múltiplas e distais, necessidade de endarterectomia, calcificação coronária difusa, dentre outros fatores). Este fato pode explicar tanto a longa duração da cirurgia e a elevada taxa (cerca de 36%) de pacientes do grupo HTK que foram incluídos no presente estudo. Também é preciso enfatizar que cada anastomose proximal entre a aorta e o enxerto venoso foi realizada durante o pinçamento aórtico.

As principais limitações deste estudo unicêntrico foram sua natureza retrospectiva e o fato de os desfechos de uma amostra limitada de pacientes ter sido investigada. Apenas desfechos precoces foram analisados e nem os desfechos pós-operatórios tardios nem os resultados angiográficos foram explorados. Consequentemente, os resultados obtidos não podem ser, de forma alguma, considerados conclusivos e devem ser verificados em populações maiores de pacientes por meio de experimentos randomizados controlados.

## Conclusões

Com base nos resultados do presente estudo, não houve diferenças significativas na proteção miocárdica entre a cardioplegia com Custodiol®-HTK e a cardioplegia sanguínea. Entretanto, os desfechos de cirurgias coronárias isoladas nas quais houve necessidade de tempo de pinçamento prologado pareceram ser piores com o uso da solução Custodiol®-HTK em relação à cardioplegia sanguínea. Embora diferenças no balanço hídrico acumulado no período pós-operatório e na função renal pareçam estar envolvidas, inúmeras variáveis podem ter interferido nos desfechos, que poderiam depender de vários aspectos, tais como diferentes equipes cirúrgicas, técnicas cirúrgicas adotadas, e protocolos de manejo perioperatório, bem como de potencial variabilidade inexplicada de pacientes.
