# Supplementary material for: Solução Custodiol®-HTK versus Cardioplegia Sanguínea Gelada em Cirurgia Coronária Isolada com Tempo de Pinçamento da Aorta Prolongado: Uma Análise de Propensão Pareada
Source: Arq Bras Cardiol. 2020 Aug 19;115(2):241–50. [Article in Portuguese] doi: 10.36660/abc.20190267 (PMC8384279; doi:10.36660/abc.20190267)
Supplement: Translation

# Translation: Custodiol®-HTK Solution vs. Cold Blood Cardioplegia for Isolated Coronary Surgery Requiring Prolonged Cross-Clamp Time: A Propensity-Matched Analysis

**Authors:** Giuseppe Gatti, Pierpaolo Taffarello, Gabriella Forti, Carla Gripari, Gianfranco Gustin, Gianluca Castaldi, Ilaria Fiorica, Aniello Pappalardo

**Affiliations:** 1 Italy Università degli Studi di Trieste, Trieste – Italy

**Keywords:** Myocardial Revascularization/complications, Heart Arrest Induced, Cardioplegic Solutions/thertapeutic use, Myocardial Reperfusion, Postoperative complications, Myocardial Infarction, Stroke, Mortality

## Abstract

**Background:**

Custodiol®-histidine-tryptophan-ketoglutarate (HTK) cardioplegia is widely used.

**Objective:**

To compare the outcomes of isolated coronary surgery requiring prolonged cross-clamp time (XCT) in patients receiving a single-dose HTK or multidose cold blood (MCB) cardioplegia.

**Methods:**

XCT was ≥120 minutes for 148 consecutive patients undergoing isolated coronary surgery (2009−2016). HTK and MCB cardioplegia were used in 38 and 110 cases, respectively. The two cohorts were compared on baseline characteristics, operative data, and early outcomes. Because risk profile and operative data differed significantly between the two groups, one-to-one propensity score-matched analysis was performed and 34 pairs were generated.

**Results:**

While expected operative risk was higher in the HTK than in the MCB cohort (European System for Cardiac Operative Risk Evaluation II, p=0.005), there was no significant intergroup difference regarding in-hospital mortality (p=0.573). Overall (positive) postoperative fluid balance (p=0.003), number of blood transfusions (p=0.017), rates of acute kidney injury (p=0.002) and any major complication (p=0.019) were increased in HTK patients. These results were all confirmed even after propensity matching, though the difference was significant only for fluid balance (p=0.013) and quite significant for blood transfusions (p=0.054). In the HTK cohort, intensive care unit and hospital stay were longer both for overall (p=0.016 and 0.008) and matched patients (p=0.142 and 0.198). In matched patients, peak serum levels of cardiac troponin I were lower in the HTK cohort (p=0.122); serum levels of creatinine were lower in the MCB cohort (p=0.023).

**Conclusion:**

For the patients of this study who required prolonged XCT, there was a trend towards poorer outcomes when HTK, rather than MCB cardioplegia, was used. (Arq Bras Cardiol. 2020; 115(2):241-250)

## Introduction

The Custodiol® histidine-tryptophan-ketoglutarate (HTK) solution (Essential Pharma, Newtown, PA, US) is classified as an intracellular, crystalloid cardioplegia because of its low sodium and calcium content.^1^ Sodium depletion of the extracellular space causes hyperpolarization of the myocardial cell plasma membrane, inducing cardiac arrest in diastole. A high histidine content buffers the acidosis caused by the accumulation of anaerobic metabolites during the ischemic period; ketoglutarate improves adenosine triphosphate production during reperfusion; tryptophan stabilizes the cell membrane.^2^ A single-dose of cardioplegia is claimed to offer myocardial protection for a period of up to three hours.^2 , 3^ Consequently, it is generally used for myocardial protection in complex, adult^4 , 5^ or pediatric cardiac operations^6^ and for heart preservation in transplant surgery.^7 , 8^ Actually, safe aortic cross-clamping time (XCT) using Custodiol-HTK cardioplegia has not been established yet. There are also concerns about hyponatremia that follows the rapid administration of the required high volume of this low-sodium cardioplegic solution,^9 , 10^ as well as the adequacy of myocardial protection offered by only a single dose of cardioplegia.^7 , 8 , 11^ In addition, despite its widespread use, very few clinical studies have compared Custodiol-HTK solution with conventional, blood or crystalloid cardioplegia in coronary bypass surgery.^12^

The aim of this retrospective study was to compare Custodiol-HTK solution with conventional multidose cold blood (MCB) cardioplegia in patients undergoing isolated coronary surgery requiring prolonged XCT. Both myocardial protection and early outcome after surgery were reviewed.

## Methods

Between July 2009 and October 2016, Custodiol-HTK cardioplegia was used in 106 consecutive patients who underwent isolated coronary artery bypass grafting at the Division of Cardiac Surgery of the University Hospital of Trieste, Italy; for 38 (35.8%) of them, XCT was of 120 minutes or longer. The early postoperative outcomes of these 38 patients (HTK cohort) were compared with those of 110 consecutive coronary surgery patients who were operated on in Trieste during the same period, had XCT ≥120 minutes, and received MCB cardioplegia (MCB cohort). Because the two cohorts differed significantly in risk profile and operative data, a propensity-matched analysis was performed as well.

Unless otherwise stated, the definitions and cut-off values of the preoperative variables were those used for the European System for Cardiac Operative Risk Evaluation II (EuroSCORE II).^13^ The risk profile of each patient was established preoperatively according to EuroSCORE II. The definitions of postoperative complications were in accordance with the internationally agreed definitions of complications after cardiac surgery.^14^ Permanent neurological dysfunction (stroke with focal lesion by computed tomography examination), prolonged (>48 h) invasive ventilation, myocardial infarction, low cardiac output (requiring high doses of inotropic agents, intra-aortic balloon pumping or use of extracorporeal membrane oxygenator), acute kidney injury (with or without renal replacement therapy), multiorgan failure, multiple blood transfusion (three red blood cells units or more), mediastinal re-exploration for bleeding or tamponade, deep sternal wound infection (deep incisional infection or mediastinitis) and sepsis were defined as major complications. In-hospital death and major complications were included in a combined endpoint.

### Cardioplegia delivering protocols

Either HTK or MCB cardioplegia was always delivered in both antegrade and retrograde mode at a temperature of ~4°C. Each patient received 20-25 ml of HTK solution per kg of body weight. The pressure of perfusion in aortic root was strictly maintained at 100 mmHg until cardiac arrest, and then at 40–50 mmHg. During the retrograde perfusion, the coronary sinus pressure was 20-25 mmHg. The cumulative perfusion time was 6-8 minutes. When hyponatremia occurred, it was corrected with sodium chloride (3 mEq/ml) solution; systemic hypotension was treated using norepinephrine infusion. Hemodilution was mitigated by removing part of the HTK solution from the aortic root during the retrograde mode.^5 , 7 , 8^ Conventional (Buckberg) MCB cardioplegia was delivered every 20 minutes, according to standardized protocols.^15^

### Blood tests

Blood levels of haemoglobin, creatinine, sodium, potassium and calcium were measured early before surgery and immediately following patient admission in intensive care unit; platelet count and blood coagulation profile before and after surgery were explored as well. Serum levels of creatine kinase, creatine kinase-MB, cardiac troponin I and aspartate aminotransferase were measured during patients’ stay in ICU. The values were compared between the HTK and MCB cohorts of propensity-matched patients.

Patients were informed about the study but were not required to provide individual consent, in accordance with the Italian legislation. Although it involves human subjects, this retrospective study was not registered in a publicly accessible database.

### Statistical methods

Continuous variables with normal distribution were expressed as mean ± standard deviation and those without normal distribution, as median and the range between the first and the third quartile. Categorical variables were expressed as frequencies and percentages. Statistical comparison of baseline patient characteristics, operative data and postoperative complications was performed using the Chi-square or the Fisher’s exact test for categorical variables, and the unpaired Student’s *t* -test or the Mann-Whitney *U* -test, for continuous variables. Since the HTK and MCB cohorts significantly differed in risk profile, number of coronary anastomoses, and length of XCT, a multivariable analysis was performed using the backward stepwise logistic regression. The area under the receiver-operating characteristic curve, with a 95% confidence interval (95%CI), was used to represent the regression probabilities. To estimate the probability of a patient being assigned to either one or other group, a propensity score (PS) was calculated in a non-parsimonious way, including the following preoperative patient characteristics: age, sex, hypertension, body mass index, haemoglobin, diabetes on insulin, chronic lung disease, glomerular filtration rate as estimated by the Cockcroft-Gault formula, chronic dialysis, extracardiac arteriopathy, New York Heart Association functional class IV, Canadian Cardiovascular Society class 4 of angina, recent myocardial infarction, left main coronary artery disease, number of diseased coronary vessels, left ventricular ejection fraction, intra-aortic balloon pump use, surgical priority, expected operative risk by EuroSCORE II, number of coronary anastomoses, and length of XCT. One-to-one PS-matching was performed employing the nearest neighbour method and a caliper of 0.2 of the standard deviation of the logit of the PS. To evaluate the balance between the matched groups, the McNemar test for dichotomous variables, the Student’s *t* -test for paired samples or the Wilcoxon test for continuous variables, and the analysis of the standardized differences after matching were used. Standardized difference <10% was considered an acceptable imbalance between the treatment groups. The same tests were adopted to test differences in operative data and postoperative complications of matched groups. Two-way analysis of variance was used to observe the interaction between the type of cardioplegia and any major complication for relevant baseline characteristics among PS-matched pairs. All tests were two-sided with the p-value set at 0.05 for statistical significance. Acquisition of the data entries was performed using Microsoft Office Excel, version 2007. Data analyses were performed using the SPSS software package for Windows, version 13.0 (SPSS, Inc., Chicago, IL, USA).

## Results

### Overall series

Although the HTK and MCB cohorts differed significantly in expected operative risk by EuroSCORE II (p=0.005), number of coronary anastomoses (p=0.003) and length of XCT (p=0.031), there was no statistically significant difference regardingt in-hospital mortality (p=0.573). However, overall (positive) postoperative fluid balance (p=0.003), acute kidney injury (p=0.002), number of transfused red blood cells units (p=0.017), and overall major complication rate (p=0.019) were all significantly increased in HTK-patients, who consequently experienced longer in-hospital stays (p=0.008) ( Tables 1-3 ).

**Table 1:** – Baseline characteristics of patients and risk profiles*,†

Characteristic	Overall series				PS-matched pairs			

	HTK cohort n=38	MCB cohort n=110	p-Value	Standardized difference	HTK cohort n=34	MCB cohort n=34	p-Value	Standardized difference
Age (years)	66±9.5	66.3±9	0.850		66.1±10	65.3±9.6	0.740	
70–80	12 (31.6)	40 (36.4)		0.001	12 (35.3)	12 (35.3)		0.027
>80	2 (5.3)	5 (4.5)		0.018	2 (5.9)	1 (2.9)		0.010
Female sex	4 (10.5)	11 (10)	1.000	0.057	2 (5.9)	1 (2.9)	1.000	0.001
Hypertension	32 (84.2)	85 (77.3)	0.489	0.007	28 (82.4)	24 (70.6)	0.252	0.000
Body mass index, kg/m^2^	28±5.1	27.6±3.7	0.582		28.2±5.1	27.2±3.1	0.318	
>30	10 (26.3)	26 (23.6)		0.008	9 (26.5)	9 (26.5)		0.001
Anaemia‡	18 (47.4)	43 (39.1)	0.371	0.010	16 (47.1)	18 (52.9)	0.624	0.000
Diabetes on insulin	5 (13.1)	7 (6.4)	0.298	0.007	3 (8.8)	5 (14.7)	0.709	0.003
Chronic lung disease	4 (10.5)	8 (7.3)	0.731	0.018	3 (8.8)	3 (8.8)	1.000	0.005
eGFR, ml/min§	75.1±39	83.5±28.4	0.156		74.9±40.9	78.6±26.7	0.659	
50–85	12 (31.6)	44 (40)		0.032	10 (29.4)	13 (38.2)		0.001
≤50	10 (26.3)	11 (10)		0.018	9 (26.5)	6 (17.6)		0.003
Chronic dialysis	2 (5.3)	2 (1.8)	0.573	0.131	2 (5.9)	1 (2.9)	1.000	0.014
Extracardiac arteriopathy	13 (34.2)	27 (24.5)	0.247	0.015	12 (35.3)	10 (29.4)	0.603	0.001
NYHA class IV	2 (5.3)	4 (3.6)	1.000	0.092	2 (5.9)	3 (8.8)	1.000	0.012
CCS class 4	24 (63.2)	52 (47.3)	0.091	0.026	20 (58.8)	18 (52.9)	0.624	0.002
Recent myocardial infarction	7 (18.4)	21 (19.1)	0.920	0.034	6 (17.6)	5 (14.7)	0.740	0.006
Coronary artery disease			0.423				0.606	
Two-vessel	1 (2.6)	9 (8.2)		0.084	1 (2.9)	3 (8.8)		0.004
Three-vessel	37 (97.4)	101 (91.8)		0.008	33 (97.1)	31 (91.2)		0.000
Left main disease	10 (26.3)	43 (39.1)	0.156	0.042	10 (29.4)	10 (29.4)	1.000	0.006
LV ejection fraction, %	52.2±13	54.9 ± 10.1	0.205		52±12.7	52.1±11.4	0.960	
31–50	13 (34.2)	30 (27.3)		0.012	11 (32.4)	13 (38.2)		0.001
<31	2 (5.3)	4 (3.6)		0.060	2 (5.9)	2 (5.9)		0.008
Use of IABP	5 (13.1)	11 (10)	0.762	0.016	3 (8.8)	2 (5.9)	1.000	0.003
Surgical priority			0.275				0.858	
Elective	8 (21.1)	37 (33.6)		0.024	8 (23.5)	10 (29.4)		0.006
Urgent	29 (76.3)	72 (65.5)		0.031	25 (73.5)	23 (67.6)		0.003
Emergency	1 (2.6)	1 (0.9)		-	1 (2.9)	1 (2.9)		-
Expected operative risk	3.5 (1.6−6.2)	1.7 (1.1−3.9)	0.005		3.3 (1.5−5.8)	3 (1.4−4.8)	0.812	
(EuroSCORE II), %||								

*Continuous variables were expressed as mean ± SD, or median and the range between the first and the third quartile. Categorical variables were expressed as frequencies and percentages. †Unless otherwise stated, the definitions and cut-off values of the preoperative variables were those used for EuroSCORE II. ‡Defined as haemoglobin <13 g/dl for men and <12 g/dl for women. §The creatinine clearance rate, calculated according to the Cockcroft-Gault formula, was used for approximating the GFR. ||Ref. 13. CCS: Canadian Cardiovascular Society; eGFR: estimated glomerular filtration rate; EuroSCORE: European System for Cardiac Operative Risk Evaluation; HYK: histidine-tryptophan-ketoglutarate; IABP: intra-aortic balloon pump; LV: left ventricular; MCB: multidose cold blood; NYHA: New York Heart Association; PS: propensity score; SD: standard deviation.

### PS-matched pairs

A PS was estimated by logistic regression and its area under the receiver-operating characteristic curve was of 0.75 (95 %CI, 0.67–0.81). One-to-one matching resulted in 34 pairs of HTK/MCB patients with similar baseline characteristics, risk profile and operative data, as confirmed by standardized differences being all lower than 10% (Tables 1 and 2). Overall (positive) postoperative fluid balance was significantly higher for HTK than for MCB patients (p=0.013) ( Table 2 ). In the HTK cohort, there was a trend towards a higher number of transfused red blood cells units (p=0.054), an increased risk of acute kidney injury (p=0.150) and any major complication (p=0.128), as well as towards longer intensive care unit (p=0.142) and in-hospital stay (p=0.198) ( Table 3 ). Test for interaction showed that the use of Custodiol-HTK solution in patients with renal impairment could increase the risk of any major complication after surgery (p=0.183; Table 4 ). Between the two cohorts, there were no significant differences in changes in blood levels of hemoglobin, platelets and electrolytes. Similarly, no significant changes were observed in the coagulation profile between the values immediately before surgery and those immediately following patient admission to ICU, whereas differences in creatinine levels were significant (p=0.023; Fig. 1 ). Finally, peak serum levels of cardiac troponin I were lower in the HTK than in the MCB cohort, though the difference was not quite significant (p=0.122; Fig. 2 ).

**Table 2:** – Operative data*

Data	Overall series				PS-matched pairs			

	HTK cohort n=38	MCB cohort n=110	p-Value	Standardized difference	HTK cohort n=34	MCB cohort n=34	p-Value	Standardized difference
No. of coronary anastomoses	5.2±1.3	4.6±1	0.003		5.1±1	5.2±1	0.481	
Grafting			0.249				0.510	
Bilateral ITA	30 (78.9)	97 (88.2)			27 (79.4)	30 (88.2)		
Single ITA	8 (21.1)	12 (10.9)			7 (20.6)	4 (11.8)		
SVGs alone	0	1 (0.9)			0	0		
XCT, min	136 (128−148)	130 (124−139)	0.031		137 (128−148)	135.5 (125.5−145.5)	0.971	
120–149	29 (76.3)	97 (88.2)		0.001	26 (76.5)	27 (79.4)		0.000
150–179	8 (21.1)	10 (9.1)		0.022	8 (23.5)	5 (14.7)		0.002
180–209	0	3 (2.7)		0.050	0	2 (5.9)		0.007
>209	1 (2.6)	0		-	0	0		-
Spontaneous VT/VF after	2 (5.3)	12 (10.9)	0.362		0	0	-	
cross-clamp releasing								
CPB time (min)	234 (217−259)	225 (209−253)	0.193		224.5 (216−252)	234.5 (222−261.5)	0.464	
Length of operation (min)	345 (326−389)	340 (315−369)	0.412		341 (326−378.5)	338 (320−366.5)	0.962	
Total fluid balance/BSA (ml/m^2^)	+2900 (2440−3590)	+2440 (2030−3140)	0.003		+2860 (2440−3660)	+2500 (2000−3030)	0.013	

*Continuous variables were expressed as mean ± SD, or median and the range between the first and the third quartile. Categorical variables were expressed as frequencies and percentages. BSA: body surface area; CPB: cardiopulmonary bypass; HYK: histidine-tryptophan-ketoglutarate; ITA: internal thoracic artery; MCB: multidose cold blood; PS: propensity score; SD: standard deviation; SVG: saphenous vein graft; VT/VF: ventricular tachycardia/fibrillation; XCT: cross-clamping time.

**Table 3:** – In-hospital outcomes*,†

Complication	Overall series			PS-matched pairs		

	HTK cohort n=38	MCB cohort n=110	p-Value	HTK cohort n=34	MCB cohort n=34	p-Value
In-hospital/30-day death	2 (5.3)	2 (1.8)	0.573	1 (2.9)	0	1.000
Neurological dysfunction (any)	2 (5.3)	8 (7.3)	1.000	2 (5.9)	5 (14.7)	0.427
Transitory‡	1 (2.6)	8 (7.3)	0.448	1 (2.9)	5 (14.7)	0.197
Permanent (stroke)	1 (2.6)	2 (1.8)	1.000	0	1 (2.9)	1.000
Prolonged (>48 h) invasive ventilation	3 (7.9)	6 (5.5)	0.695	2 (5.9)	3 (8.8)	1.000
Atrial fibrillation, new-onset	6 (15.8)	16 (14.5)	0.862	5 (14.7)	4 (11.8)	1.000
Myocardial infarction	0	1 (0.9)	1.000	0	1 (2.9)	1.000
RV dysfunction	4 (10.5)	8 (7.3)	0.731	4 (11.8)	1 (2.9)	0.356
Low cardiac output	6 (15.8)	10 (9.1)	0.362	3 (8.8)	3 (8.8)	1.000
Prolonged (>12 h) inotropic support	23 (60.5)	57 (51.8)	0.354	19 (55.9)	19 (55.9)	1.000
Intra- and postoperative use of IABP	0	9 (8.2)	0.112	0	3 (8.8)	0.239
Acute kidney injury	8 (21.1)	4 (3.6)	0.002	7 (20.6)	2 (5.9)	0.150
Renal replacement therapy	5 (13.2)	0	0.001	4 (11.8)	0	0.114
Multiorgan failure	3 (7.9)	3 (2.7)	0.339	2 (5.9)	1 (2.9)	1.000
48-h Chest tube output/BSA, ml/m2	685 (390–1074.5)	551 (372.5–970)	0.463	616.5 (390–1074.5)	633 (381–953.5)	0.609
No. of transfused RBC units	1.5 (1–3)	1 (0–2)	0.017	2 (1–3)	1 (0–2)	0.054
Multiple blood transfusion (>2 RBC units)	12 (31.6)	19 (17.3)	0.062	11 (32.4)	7 (20.6)	0.271
Massive blood transfusion (>4 RBC units)	5 (13.2)	2 (1.8)	0.012	4 (11.8)	0	0.114
Mediastinal re-exploration	4 (10.5)	6 (5.5)	0.454	3 (8.8)	2 (5.9)	1.000
Deep sternal wound infection	1 (2.6)	4 (3.6)	1.000	0	1 (2.9)	1.000
Sepsis	0	2 (1.8)	1.000	0	1 (2.9)	1.000
Any major complication§	17 (44.7)	27 (24.5)	0.019	15 (44.1)	9 (26.5)	0.128
In-hospital stay (days)	13.5 (10–18)	10 (8–15)	0.008	14 (10–19)	10 (8–15)	0.198
Intensive care unit stay (days)	3 (2–5.5)	2 (1.5–3)	0.016	3 (2–5.5)	2 (1.5–3)	0.142

*Continuous variables were expressed as median and the range between the first and the third quartile. Categorical variables were expressed as frequencies and percentages. †Unless otherwise stated, the definitions of the postoperative complications were in accordance with the internationally agreed definitions of complications after cardiac surgery. ‡Including delayed awakening, manifest psychiatric disorder and seizures. §Including in-hospital death, stroke, prolonged invasive ventilation, myocardial infarction, low cardiac output, acute kidney injury, multiorgan failure, multiple blood transfusion, mediastinal re-exploration, deep sternal wound infection and sepsis. BSA: body surface area; HYK: histidine-tryptophan-ketoglutarate; IABP: intra-aortic balloon pump; MCB: multidose cold blood; PS: propensity score; RBC: red blood cells; RV: right ventricular.

**Table 4:** – Analysis of the rate of any major complication post-surgery* in different subgroups of patients with testing for interaction†

Characteristic	HTK cohort %	MCB cohort %	OR	95% CI	Interaction

					p-Value
Overall	44.1	26.5	2.19	0.79−6.08	0.132
No anaemia	33.3	25	1.5	0.34–6.7	0.391
Anaemia‡	56.2	27.8	3.34	0.8–13.9	
eGFR >85 ml/min§	33.3	33.3	1.00	0.22–4.56	0.183
eGFR ≤85 ml/min§	52.6	21.1	4.17	1–17.3	
CCS class 1–3	50	31.2	2.2	0.5–9.75	0.970
CCS class 4	40	22.2	2.33	0.56–9.72	
Recent myocardial infarction	46.4	27.6	2.27	0.76–6.85	0.864
No recent myocardial infarction	33.3	20	2	0.13–32	
LV ejection fraction >50%	38.1	26.3	1.72	0.45–6.64	0.522
LV ejection fraction ≤50%	53.8	26.7	3.21	0.66–15.6	

*Including in-hospital death, stroke, prolonged invasive ventilation, myocardial infarction, low cardiac output, acute kidney injury, multiorgan failure, multiple blood transfusion, mediastinal re-exploration, deep sternal wound infection and sepsis. †Unless otherwise stated, the definitions and cut-off values of the preoperative variables were those used for EuroSCORE II. ‡Defined as haemoglobin <13 g/dl for men and <12 g/dl for women. §The creatinine clearance rate, calculated according to the Cockcroft-Gault formula, was used for approximating the GFR. CCS: Canadian Cardiovascular Society; CI: confidence interval; eGFR: estimated glomerular filtration rate; EuroSCORE: European System for Cardiac Operative Risk Evaluation; HYK=histidine-tryptophan-ketoglutarate; LV: left ventricular; MCB: multidose cold blood; OR: odds ratio.

**Figure 1A:** – *HTK vs. MCB-cohort. PS-matched pairs. Differences on changes of blood levels of haemoglobin, platelets, INR and aPTT between the values early before surgery and the values immediately following patient admission to intensive care unit. aPTT: activated partial thromboplastin time; HTK: histidine-tryptophan-ketoglutarate; INR: international normalized ratio; MCB: multidose cold blood; PS: propensity score.*

**Figure 1B:** – *HTK vs. MCB cohort. PS-matched pairs. Differences on changes of blood levels of creatinine, sodium, potassium and calcium between the values early before surgery and the values immediately following patient admission to intensive care unit. HTK: histidine-tryptophan-ketoglutarate; MCB: multidose cold blood; PS: propensity score.*

**Figure 2:** – *HTK vs. MCB cohort. PS-matched pairs. Differences on peak serum levels after surgery of CK-MB, CK-MB/CK, cTnI and AST. AST: aspartate aminotransferase; CK: creatine kinase; CK-MB: creatine kinase-MB; cTnI: cardiac troponin I; HTK: histidine-tryptophan-ketoglutarate; MCB: multidose cold blood; PS: propensity score.*

## Discussion

The most relevant finding of this study was that, with respect to conventional MCB cardioplegia, Custodiol-HTK solution did not improve clinical outcomes of a limited series of patients undergoing isolated coronary operations requiring XCT of 120 minutes or longer. In effect, after propensity matching, higher blood levels of creatinine were observed in HTK patients early after surgery, as well as a trend towards a higher number of transfused red blood cells units, an increased risk of acute kidney injury (and renal replacement therapy) and any major complication, and longer intensive care unit and hospital stays. In addition, the test for interaction showed that the use of Custodiol-HTK solution in patients with renal impairment could increase the risk of any major complication after surgery. However, the poorer performance of this intracellular cardioplegia in the difficult subset of patients of the present study was not related to inadequate myocardial protection. Actually, between the HTK and MCB cohorts there were differences neither in the rates of myocardial infarction and low cardiac output nor in the rates of their surrogates, such as prolonged inotropic support, intraoperative and postoperative use of IABP. Besides, the peak levels of cardiac troponin I tended to be lower in HTK than in MCB patients. The increased need for red blood cells units, primarily during surgery, in the HTK cohort was mainly due to hemodilution, associated with administration of large volumes of crystalloid cardioplegia, as required by this method. Despite removing part of the HTK solution from the aortic root during the retrograde mode of delivering, overall (positive) postoperative fluid balance was indeed significantly higher for HTK than MCB patients, even after propensity-matched analysis. In fact, there was neither increased bleeding post-surgery nor any difference on blood coagulation profiles in HTK patients compared with MCB patients. Both hemodilution and the following need for transfusions that occurred in the HTK cohort during operation, as well as the increased positive fluid balance that ensued, could explain both the increased rate of acute kidney injury (and renal replacement therapy) in these patients and the increased risk of any major postoperative complication in HTK patients with renal impairment. Certainly, some systemic metabolic effects might be even involved. For instance, some grade of metabolic acidosis occurs frequently following the use of Custodiol-HTK solution, and must be promptly neutralized.^1 - 3^ However, since no perioperative data on pH or lactacidemia have been reported, this hypothesis could not be confirmed in the study. On the other hand, because the Custodiol-HTK solution is being successfully used to preserve renal function in kidney transplantation surgery^16^ , direct kidney injury seems unlikely.

Although there was an evident difference in in-hospital mortality between the two study groups (5.6% vs. 1.8%), this difference was not significant (p=0.573), maybe owing to the limited number of study patients. However, there was no difference in in-hospital mortality (p=1.000) after propensity matching, which compensated for difference in expected operative risk in the overall cohort. No increased rate of spontaneous ventricular tachycardia or fibrillation after cross-clamp releasing was recorded. No significantly increased risk of right ventricular dysfunction was reported. No significant benefit derived from using either Custodiol-HTK or MCB cardioplegia for patients with anaemia, unstable angina, recent myocardial infarction or left ventricular dysfunction. The perioperative strict and effective control of hyponatremia, which was obtained in the study patients, could explain the low rate of any transitory neurological dysfunction that occurred in HTK cohort. The lower rate of any transitory neurological dysfunctions observed in HTK patients compared to the MCB group (albeit the difference was not quite significant) was an unexpected result for the present authors.

The role of Custodiol-HTK solution in adult cardiac surgery has not been explored in depth. Generally, the authors who have investigated outcomes following minimally invasive cardiac surgery using Custodiol-HTK solution agreed that avoiding repetitive infusions may reduce the risk for coronary malperfusion due to dislodgement of the endoaortic clamp (if used) and increase the surgeon’s comfort during the procedure.^17 - 20^ Almost all of the investigators who have compared Custodiol-HTK with cold blood cardioplegia have shown similar clinical outcomes for the two options.^5 , 7 , 8 , 17 , 19^ Actually, only few studies have shown some benefits deriving from either one of the two cardioplegic strategies. For example, lower values of cardiac troponin I for XCT >160 minutes have been reported for Custodiol-HTK patients undergoing aortic surgery by Scrascia et al.^4^ . Prathanee et al.^12^ have stigmatized that using Custodiol-HTK cardioplegia in isolated coronary surgery leads to a significantly increased risk of spontaneous ventricular fibrillation after releasing of the aortic clamp. However, no clinical significance has been given to this fact. Very recently, in a series of 362 patients who underwent (minimally invasive or open) heart valve surgery, Hummel et al.^20^ have shown superior outcomes for Custodiol-HTK patients as to blood transfusion, stroke and 30-day hospital readmission from discharge, this translating in an average hospital net savings of about $3,000 per patient. Finally, equivalent outcomes between Custodiol-HTK and cold blood cardioplegia have been shown by Hoyer et al.^11^ for 825 pairs of propensity score-matched patients, even though blood cardioplegia seemed to be beneficial in the presence of left ventricular dysfunction.

Because a single-dose of Custodiol-HTK cardioplegia is claimed to offer prolonged myocardial protection,^2 , 3^ during the study period it was generally used, in coronary surgery, for patients with expected long time of surgery (high number of coronary anastomoses, low diameter of the coronary vessels, intramyocardial course, multiple and distal lesions, need for endarterectomy, diffuse coronary calcification, …). This fact may explain both the very long operation times and the high rate (about 36%) of HTK patients who were enrolled in the present study. It should also be emphasized that every proximal anastomosis between the aorta and venous graft was performed during aortic cross-clamping.

The primary limitations of this single-center study were its retrospective nature and the fact that outcomes of a limited series of patients were investigated. Only early outcomes were reviewed and neither late outcomes post-surgery nor coronary angiographic results were explored. Consequently, the results obtained can in no way be considered conclusive and should be verified in larger patient populations by means of randomized controlled trials.

## Conclusions

Based on the results of the present study, there was no significant difference in myocardial protection between Custodiol-HTK and MCB cardioplegia. However, outcomes of isolated coronary surgery requiring prolonged XCT seemed to be poorer using Custodiol-HTK solution rather than MCB cardioplegia. Although differences in postoperative fluid balance and renal function seemed to be involved, several variables might have interfered in the outcomes, which could depend from a number of aspects related to different surgical teams, adopted surgical techniques, and protocols of perioperative management, as well as from a potential unexplained variability of patients.
